# Impact of postoperative major complications on long-term survival after radical resection of gastric cancer

**DOI:** 10.1186/s12885-019-6024-3

**Published:** 2019-08-23

**Authors:** Peng Yuan, Zhouqiao Wu, Ziyu Li, Zhaode Bu, Aiwen Wu, Xiaojiang Wu, Lianhai Zhang, Jinyao Shi, Jiafu Ji

**Affiliations:** 1 Key Laboratory of Carcinogenesis and Translational Research (Ministry of Education), Department of Endoscopy Center, Peking University Cancer Hospital & Institute, #52, Fucheng Road, Haidian, Beijing, People’s Republic of China; 2 Key Laboratory of Carcinogenesis and Translational Research (Ministry of Education), Department of Gastrointestinal Cancer Center Surgery, Peking University Cancer Hospital & Institute, #52, Fucheng Road, Haidian, Beijing, People’s Republic of China

**Keywords:** Gastric cancer, Complications, Clavien–Dindo classification, Survival

## Abstract

**Background:**

This study was designed to evaluate the impact of postoperative major complications on long-term survival following curative gastrectomy.

**Methods:**

This retrospective study included 239 patients with gastric cancer undergoing gastrectomy at the Beijing Cancer Hospital from February 2012 to January 2013. Survival curves were compared between patients with major complications (mC group) and those without major complications (NmC group). Multivariate analysis was conducted to identify independent prognostic factors.

**Results:**

Postoperative complication and mortality rates were 24.7 and 0.8%, respectively. The severity of complications was graded in accordance with the Clavien–Dindo classification. The incidence of minor complications (grades I-II) and major complications (grades III–V) was 9.2 and 15.5%, respectively. The 3-year overall survival (OS) and disease-free survival (DFS) rates were better in the NmC group than in the mC group (*p* = 0.014, *p* = 0.013). Multivariate analysis identified major complications as an independent prognostic factor for OS and DFS. After stratification by pathological stage, this trend was also observed in stage II patients.

**Conclusions:**

Postoperative major complications adversely affect OS and DFS. The prevention and early diagnosis of complications are essential to minimize the negative effects of complications on surgical safety and long-term patient survival.

## Background

Recent developments in the field of anti-cancer treatment have decreased cancer-related mortality [1]. Surgical resection remains the gold-standard treatment for gastric cancer. In the era of modern surgery, perioperative mortality and reoperation rates after gastric cancer have decreased to low levels [2]. However, the rate of postoperative complications remains unsatisfactory, varying from 18.3 to 36% [3–5]. Postoperative complications, particularly surgical complications, significantly prolong hospital stay and medical expenditure and have become major causes of postoperative short-term mortality.

Recent data have suggested that patients who suffer from complications have poorer long-term survival outcomes. The underlying reason for this trend remains to be established [6–9]. A major limitation of previous study is that most of those data were retrospectively collected, and complications are often diagnosed by different doctors. Such heterogeneity inevitably increases the amount of record bias, which may influence the analysis. Another pitfall is the failure of attending physicians to evaluate the severity of postoperative complications, which compromises a detailed analysis of the results. To this end, we analyzed data collected at our institution for cases seen during the period from 2012 to 2013. During that period, all clinical complication data were recorded and classified by a single doctor (chief resident) who was familiar with all cases and responsible for the quality control of medical records during that period. All data about treatment decision making was validated by the Multidisciplinary team (MDT) every Friday morning. The aim of this study was to investigate the impact of postoperative complications on long-term survival after radical resection of gastric cancer, to evaluate the severity of complications according to the Clavien–Dindo scoring system, and analyze the impact of major and minor complications.

## Methods

This is a retrospective study that investigated the effects of surgical complications. We enrolled 239 consecutive patients who had previously undergone gastrectomy with D2 lymph node dissection at the Beijing Cancer Hospital from February 2012 to January 2013. All patients recruited to our study were diagnosed with gastric adenocarcinoma, as confirmed by endoscopy and histology. Patients with any other malignancy were excluded.

Patient characteristics and clinical records were obtained from the HIS medical system at our institution. For each patient, the following clinical data were collected and analyzed: patient factors (age, sex, Body mass index (BMI), comorbidity, and prior history of abdominal surgery), tumor pathology, treatment factors (operation time, combined organ resection, blood loss, preoperative chemotherapy, extent of resection, and surgical type), and the length of hospital stay. Tumors were staged in accordance with the Union for International Cancer Control Classification System (7th edition). According to this system, patients receiving neo-adjuvant chemotherapy who respond with a complete pathological response following surgery are classified as stage 0.

Postoperative outcome and details of any resulting complications were also collected from the electronic medical records. The complications recorded and corresponding diagnostic criteria are listed in Table 1. All complications are categorized according to the Clavien–Dindo classification system [10]. If a patient had more than one complication, then the grade used for analysis was determined by the highest ranked complication. Complications of grades I-II were defined as minor complications, whereas those of grades III–V were considered as major complications.

**Table 1:** Complications and the corresponding diagnostic criteria

Complication	Diagnostic notes
Anastomotic leakage	Leakage confirmed by angiography or bowel content seen in drainage
Anastomotic bleeding	Continuous blood content seen in gastric tube and continuous decreasing hemoglobin level, within 24 h after surgery
Abdominal bleeding	Continuous blood content seen in drainage and continuous decreasing hemoglobin level, within 24 h after surgery
Surgical site infection	Wound abscess with clinical symptoms
Bowel obstruction	Clinical symptoms with radiographic confirmation
Abdominal effusion	Continuous high abdominal drainage output
Pancreatitis	Drain output of any measurable volume of fluid on or after postoperative day 3 with an amylase content greater than 3 times the serum amylase activity
Bowel preformation	Intraoperative or radiographic confirmation
Pneumonia	Clinical symptoms with radiographic confirmation

### Follow-up

Complete follow-up data were available for all patients followed with a standardized protocol at our institution. The duration of follow-up was calculated from the date of surgery. Follow-up data were collected from telephone records and records in the outpatient clinical database obtained after discharge. The end of the follow-up period was 3 years after surgery or death. Overall survival (OS) time was defined as the interval from the date of surgery to the date of death or that from the date of surgery to the date of the last follow-up examination for patients who remained alive. Disease-free survival (DFS) time was defined as the time period from surgery to date of relapse or distal metastasis.

### Statistical analysis

All statistical analyses were conducted using SPSS for Windows version 22.0 (Chicago, IL, USA). Categorical variables were analyzed using chi-square or Fisher’s exact test. Independent risk factors for complications were identified using binary logistic regression. The 3-year OS and DFS rates were calculated using the Kaplan–Meier method. The log-rank test was used to compare between the groups. Multivariate analyses were conducted using the Cox proportional hazards model. All tests were two-sided, and *p* < 0.05 was considered statistically significant difference.

## Results

### Patient demographics and surgical outcomes

Demographic and clinical characteristics of patients recruited in this study are shown in Table 2. The type and severity of postoperative complications are shown in Table 3. Overall, complications occurred in 59 (24.7%) cases, and the mortality rate was 0.8% (2/239). Incidences of minor (grades I-II) and major (grades III–V) complications were 9.2% (22/239) and 15.5% (37/239), respectively. The most frequent complication was anastomotic leakage (18/239, 7.5%), followed by gastric stasis (10/239, 4.2%) and abdominal bleeding (6/239, 2.5%). Twelve patients required surgical management. Two patients died from postoperative complications—one patient died at 11 days after surgery because of an uncontrollable postoperative infection, and the other patient died after 4 months following the deterioration of postoperative gastric stasis.

**Table 2:** Characteristics of 239 patients undergoing gastrectomy

Variables	Total (*n* = 239)
Age	59.0 ± 12.0
Sex	
Male	166 (69.5%)
Female	73 (30.5%)
Age	
≤ 55	71 (39.4)
> 55	109 (60.6)
BMI (kg/m2)	22.6 ± 3.4
Operation time (min)	220 ± 63.1
Resection type	
Subtotal	113 (47.3%)
Total	126 (52.7%)
Combined organ resection	
Yes	10 (4.2%)
No	229 (95.8%)
Neoadjuvant chemotherapy	
Yes	86 (36.0%)
No	153 (64.0%)
Surgery type	
Open	208 (87.0%)
Laparoscope	31 (13.0%)
Comorbidity Diabetes	
Yes	18 (7.5%)
No	221 (92.5%)
Cardiovascular Diseases	
Yes	46 (19.2%)
No	193 (80.8%)
Prior abdominal surgery	
Yes	31 (13.0%)
No	208 (87.0%)
TNM staging (AJCC 7th)	
0	6 (2.5%)
I	80 (33.5%)
II	58 (24.2%)
III	86 (36.0%)
IV	9 (3.8%)
Postoperative hospital stay (days)	13 ± 24.8 (range 6–284)

**Table 3:** Details of postoperative complications following gastrectomy

Type of complication	Grade according to the Clavien–Dindo classification							Total
	I	II	IIIa	IIIb	IVa	IVb	V	
Anastomotic leakage		2	11	3	1		1	18
Anastomotic bleeding		1	2	1				4
Abdominal bleeding		3	1	2				6
Wound infection	2	3						5
Intestinal obstruction	4			1				5
Gastric stasis		3	6				1	10
Duodenal stump leakage		1		1	1			3
Abdominal effusion			3					3
Pancreatitis				1				1
Bowel perforation				1				1
Pneumonia		3						3
Total	6	16	23	10	2	0	2	59

### Risk factors associated with complications

Patients were divided into the complication (C group) and no-complication (NC group) groups. As shown in Table 4, there was no significant difference between the C group and NC group in terms of sex, comorbidity, a prior history of abdominal surgery, preoperative chemotherapy, the extent of resection (total vs. subtotal), or the type of surgery (open vs. laparoscopic). The development of postoperative complications was associated with age > 55 years, BMI ≥ 25, operation time > 200 min, and combined organ resection (*p* < 0.05). Further multivariate analysis identified age, BMI, operation time, and combined organ resection as independent risk factors for the development of postoperative complications (Table 5).

**Table 4:** Univariate analysis for risk factors associated with complications following gastrectomy

Variables	No complication *n* = 180	Complication *n* = 59	*P* value
Sex			0.325
Male	122 (67.8)	44 (74.6)	
Female	58 (32.2)	15 (25.4)	
Age			0.029
≤ 55	71 (39.4)	14 (23.7)	
> 55	109 (60.6)	45 (76.3)	
BMI			0.027
< 25	134 (74.4)	35 (59.3)	
≥ 25	46 (25.6)	24 (40.7)	
Operation time (min)			0.018
≤ 200	80 (44.4)	16 (27.1)	
> 200	100 (55.6)	43 (72.9)	
Resection type			0.242
Subtotal	89 (49.4)	24 (40.7)	
Total	91 (50.6)	35 (59.3)	
Combined organ resection			0.016
Yes	4 (2.2)	6 (10.2)	
No	176 (97.8)	53 (89.8)	
Neoadjuvant chemotherapy			0.136
Yes	60 (33.3)	26 (44.1)	
No	120 (66.7)	33 (55.9)	
Surgery type			0.877
Open	157 (87.2)	51 (86.4)	
Laparoscope	23 (12.8)	8 (13.6)	
Comorbidity Diabetes			0.160
Yes	11 (6.1)	7 (11.9)	
No	169 (93.9)	52 (88.1)	
Cardiovascular Diseases			0.606
Yes	36 (20.0)	10 (16.9)	
No	144 (80.0)	49 (83.1)	
Prior abdominal surgery			0.135
Yes	20 (11.1)	11 (18.6)	
No	160 (88.9)	48 (81.4)

**Table 5:** Multivariate analysis for risk factors associated with complications following gastrectomy

Variables	Hazard ratio	95%CI	*P* value
Age			
≤ 55 vs. > 55	2.392	1.184–4.832	0.015
BMI			
< 25 vs. ≥25	2.124	1.110–4.062	0.023
Operation time (min)			
≤ 200 vs. > 200	2.066	1.060–4.029	0.033
Combined organ resection			
Yes vs. no	4.334	1.113–16.876	0.034

### Complication and survival

**Fig. 1:**
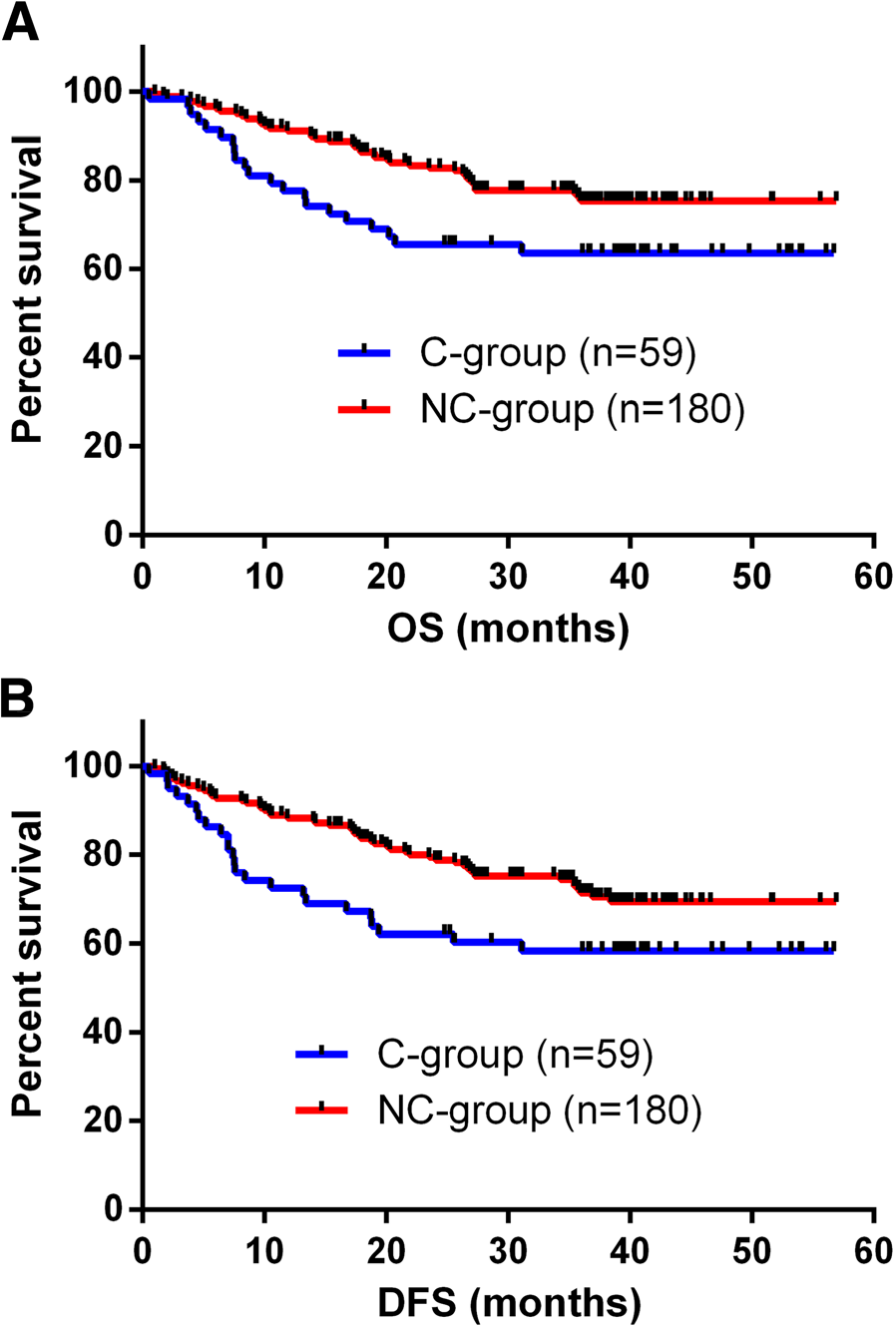
**a** Overall survival curves for 239 patients who underwent curative gastrectomy for gastric cancer. The 3-year overall survival rate is significantly better in the NC group than in the C group (*p* = 0.033). **b** Disease-free survival curves for 239 patients who underwent curative gastrectomy for gastric cancer. The 3-year disease-free survival rate is significantly better in the group of patients without complications than in the group with complications (*p* = 0.034)

Median survival during the follow-up period was 36.3 months. The 3-year OS (*p* = 0.033) and DFS (*p* = 0.034) rates were significantly better in the NC group than in the C group (Fig. 1). To determine the types of complications that had the greatest impact on survival, the effects of major (CD grade III or higher) and infectious complications were analyzed.

**Fig. 2:**
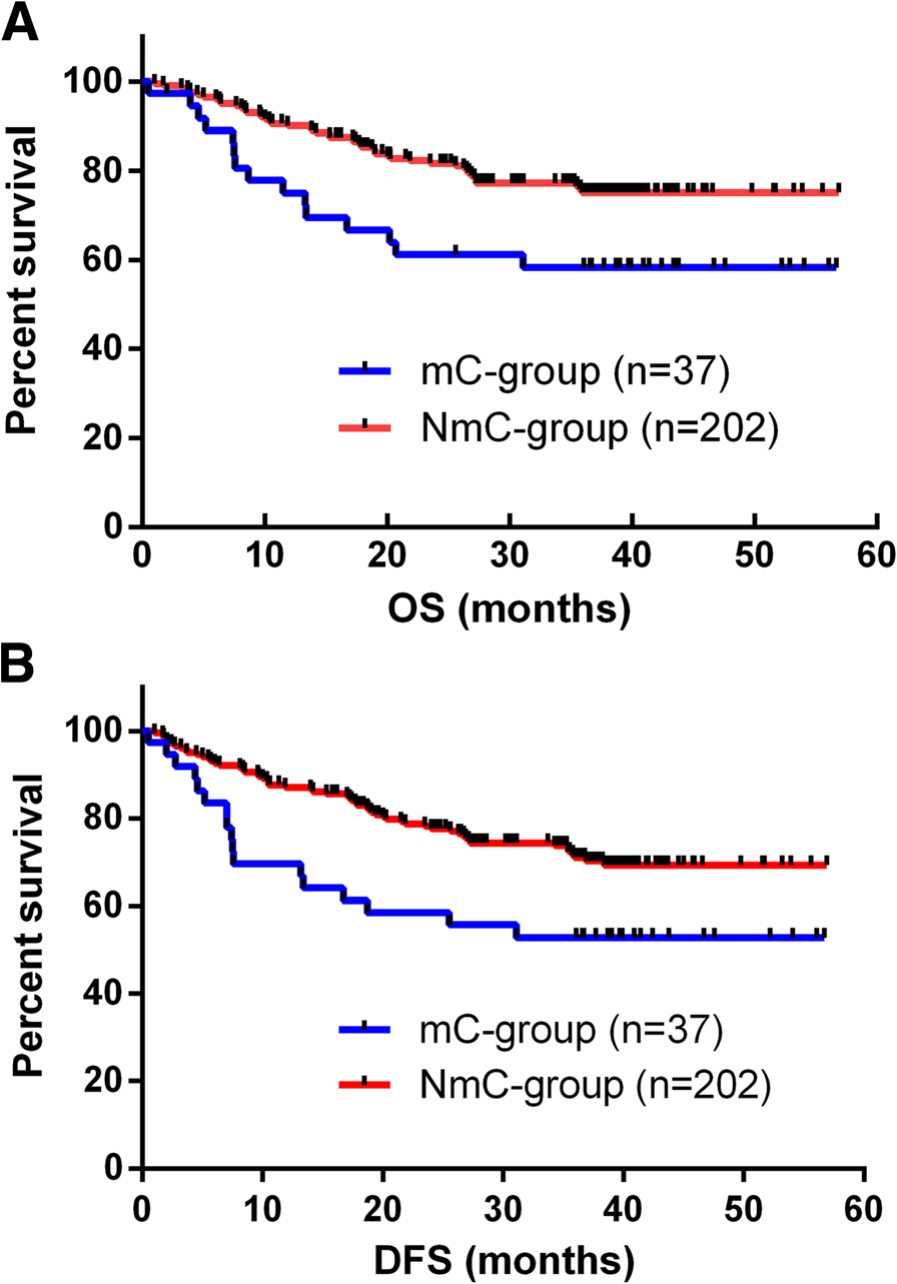
**a** Overall survival curves for 239 patients who underwent curative gastrectomy for gastric cancer. The 3-year overall survival rate was significantly better in the NmC group than in the mC group (*p* = 0.014). **b** Disease-free survival curves for 239 patients who underwent curative gastrectomy for gastric cancer. The 3-year disease-free survival rate was significantly better in the group of patients without major complications than in the group with major complications (*p* = 0.013)

In our study, major complications were those that were Clavien–Dindo grade III or higher. Patients without major complications (NmC group) had significantly better 3-year OS (*p* = 0.014) and DFS (*p* = 0.013) rates than those with major complications (Fig. 2). Results of multivariate analysis revealed that major complications and advanced TNM stage were independent risk factors for decreased 3-year OS and DFS rates in patients with gastric cancer who had undergone D2 gastrectomy (Table 6).

**Table 6:** Results of multivariate analysis to identify independent prognostic factors for overall survival and disease-free survival (major complication)

Variables	OS		*P* value	DFS		*P* value
	Hazard ratio	95%CI		Hazard ratio	95%CI	
TNM staging	3.426	2.275–5.161	< 0.001	3.315	2.330–4.717	< 0.001
Introvascular cancer emboli						
Yes vs. no	1.365	0.808–2.306	0.244	1.215	0.754–1.959	0.423
Combined organ resection						
Yes vs. no	1.431	0.625–3.275	0.397	1.531	0.687–3.410	0.298
Resection type						
Subtotal vs. Total	1.559	0.862–2.820	0.141	1.482	0.868–2.530	0.15
Major complication						
Yes vs. no	1.939	1.056–3.561	0.033	1.947	1.18–3.391	0.019

**Fig. 3:**
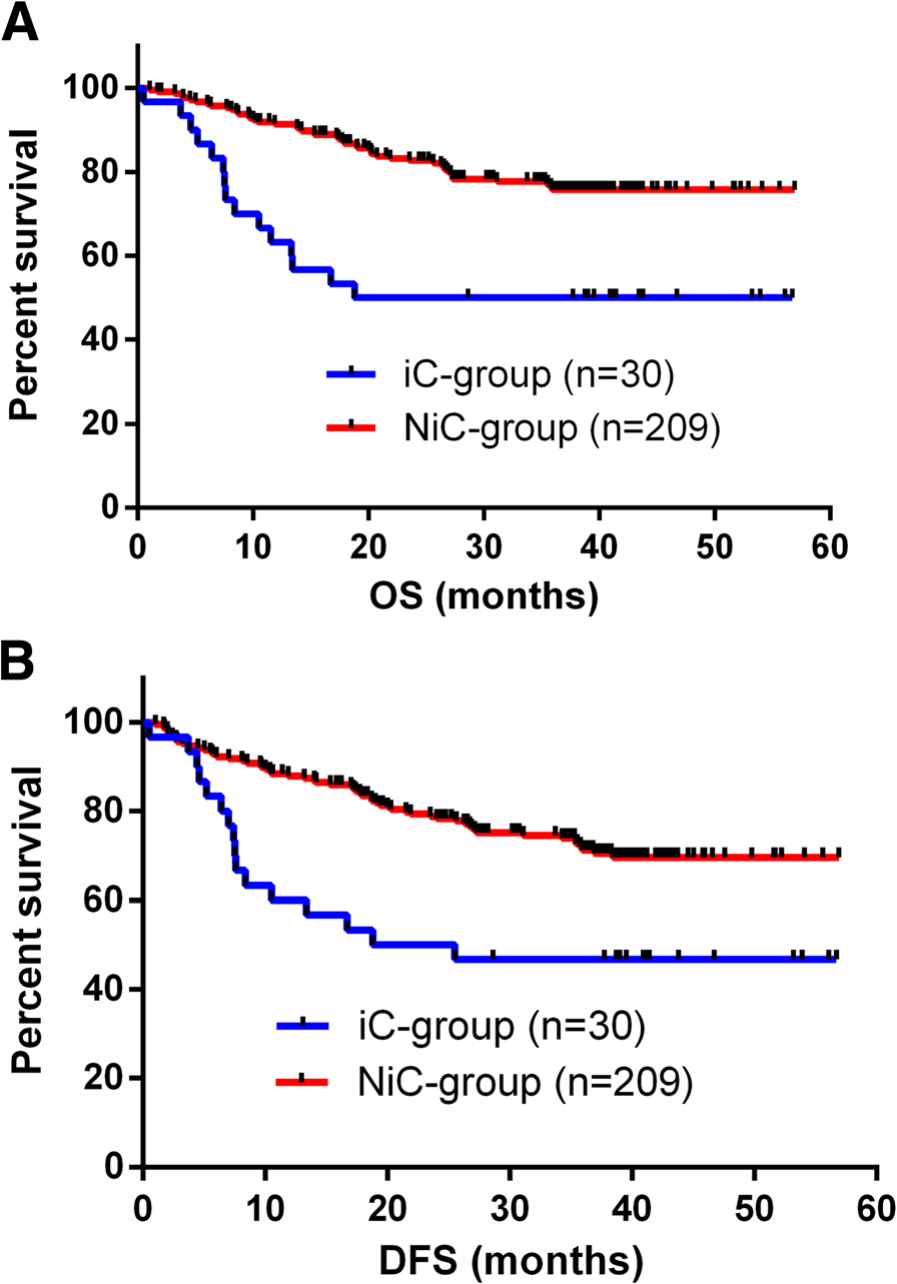
**a** Overall survival curves for 239 patients who underwent curative gastrectomy for gastric cancer. The 3-year overall survival rate was significantly better in the NiC group than in the iC group (*p* < 0.001). **b** Disease-free survival curves for 239 patients who underwent curative gastrectomy for gastric cancer. The 3-year disease-free survival rate was significantly better in the group of patients without infectious complications than in the group with infectious complications (*p* = 0.001)

This study included 30 cases of infectious complications, including 18 cases of anastomotic leakage, 3 cases of duodenal stump leakage, 5 cases of surgical site infection, 1 case of bowel perforation, and 3 cases of pneumonia. We further analyzed the data and found that patients without infectious complications (NiC group) had significantly better 3-years OS (*p <* 0.001) and DFS (*p* = 0.001), compared with patients who had infectious complications (Fig. 3). The survival analysis showed that having infectious complications and higher TNM stage were independent risk factors for poorer OS and DFS (Table 7).

**Table 7:** Results of multivariate analysis to identify independent prognostic factors for overall survival and disease-free survival (infectious complication)

Variables	OS		*P* value	DFS		*P* value
	Hazard ratio	95%CI		Hazard ratio	95%CI	
TNM staging	3.284	2.199–4.903	< 0.001	3.197	2.256–4.529	< 0.001
Introvascular cancer emboli						
Yes vs. no	1.327	0.782–2.252	0.294	1.178	0.728–1.906	0.504
Combined organ resection						
Yes vs. no	1.713	0.765–3.832	0.19	1.662	0.751–3.676	0.21
Resection type						
Subtotal vs. Total	1.464	0.802–2.671	0.214	1.449	0.844–2.488	0.179
Infectious complication						
Yes vs. no	2.47	1.346–4.533	0.004	1.935	1.083–3.457	0.026

**Fig. 4:**
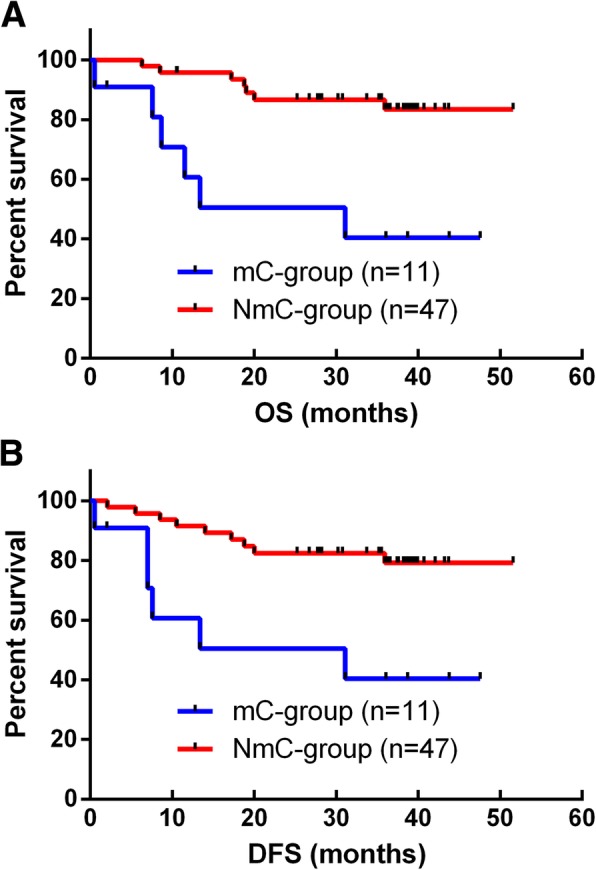
**a** Overall survival curves for 58 stage II patients who underwent curative gastrectomy for gastric cancer. The 3-year overall survival rate was significantly better in the NmC group than in the mC group (*p* = 0.001). **b** Disease-free survival curves for 58 stage II patients who underwent curative gastrectomy for gastric cancer. The 3-year disease-free survival rate was significantly better in the group of patients without major complications than in the group with major complications (*p* = 0.003)

Further subgroup analysis showed that, among stage II patients, major complications were associated with poorer 3-year OS and DFS rates (*p* = 0.001 and *p* = 0.003, respectively; Fig. 4). The multivariate analysis also identified major complications as an independent prognostic factor for OS and DFS in the subgroup of patients with stage II gastric cancer (Table 8).

**Table 8:** Results of multivariate analysis to identify independent prognostic factors for overall survival and disease-free survival in the subgroup of II stage gastric cancer

Variables	OS		*P* value	DFS		*P* value
	Hazard ratio	95%CI		Hazard ratio	95%CI	
Introvascular cancer emboli						
Yes vs. no	0.439	0.092–2.093	0.301	0.37	0.08–1.715	0.204
Combined organ resection						
Yes vs. no	1.48	0.173–12.684	0.72	1.224	0.149–10.077	0.851
Resection type						
Subtotal vs. Total	1.125	0.356–3.557	0.841	1.024	0.354–2.962	0.965
Major complication						
Yes vs. no	5.667	1.842–17.439	0.002	4.34	1.498–12.571	0.007

## Discussion

Postoperative complications, especially surgical complications, significantly prolong hospital stay and medical expenditure, and impair short-term survival after surgery, with possible negative impacts on long-term survival. In this study, we analyzed medical records for consecutive gastric cancer cases seen during the period from 2012 to 2013. We confirmed poorer long-term survival in patients with postoperative complications. Subsequent analyses confirmed that major complications and infectious complications are major causes of impaired long-term survival.

The topic of “complication and survival” has been examined repeatedly, with controversial results. Several studies have reported a correlation between postoperative complications and poor long-term outcome among patients with esophageal or gastroesophageal cancer [6, 11, 12]. Among patients with colorectal cancer, postoperative complications are associated with a higher rate of recurrence and worse long-term outcomes [13–15]. Branagan and Finnis reported that rectal anastomosis leakage, compared with colonic anastomosis leakage, increased the risk of local recurrence after colorectal surgery. However, the same authors reported no difference in long-term survival between patients with vs. without anastomotic leakage [8]. A similar result was reported by Junemann-Ramirez et al. who found that anastomotic leakage did not shorten 5-year survival in patients undergoing esophagogastrectomy, though 30-day mortality was much higher in the group with anastomotic leakage [16].

One possible explanation for this controversial data is that only severe complications (CD grade III or higher), but not minor complications (CD grade I-II), impair patient survival. We performed subgroup analysis, the results of which confirmed poorer survival in patients with major complications.

Most theories attribute poorer survival in patients with complications to the local recurrence or distant metastasis of cancer [7, 17, 18]. In patients who undergo colorectal resection, long-term outcomes may be severely impaired when anastomotic leakage occurs [14, 15]. It has been proposed that anastomotic leakage leads to the deposition and implantation of viable exfoliated tumor cells in the pelvis, which results in an increased rate of local recurrence [13]. However, in their study, Tokunaga et al. did not observe local recurrence in any patient with anastomotic leakage [7].

With regard to infectious complications such as intra-abdominal abscess, abdominal infection, and pneumonia, the poorer survival observed in our study and in previous investigations may reflect immune suppression that results in cancer recurrence and poorer survival [19, 20]. Postoperative infections induce proinflammatory cytokine cascades. Inflammatory cytokines such as tumor necrosis factor-alpha (TNF-α) and interleukins 1, 6 and 8 (IL-1/6/8) may interfere with the function of natural killer cells, cytotoxic T-lymphocytes, and antigen-presenting cells [21–23], which promote the growth and metastasis of tumor cells. Postoperative infections also delay the initial date of adjuvant chemotherapy after surgery, which may further diminish survival [24, 25].

One advantage of our study is the fact that our database is prospectively maintained. Furthermore, all data related to the incidence of complications were double checked and recorded by the chief resident, who was very familiar with the perioperative condition of each patient. The second check also minimized possible bias among researchers. After the above-mentioned efforts, our recorded complication rates were comparable to those of many high-quality databases [3–5].

Further subgroup analysis showed that major complications were an independent prognostic factor for OS and DFS in the subgroup with stage II gastric cancer. In this study, the mean duration of postoperative hospital stay was 11.9 (6–24) days among patients without postoperative complications and 38.1 (8–284) days among those with postoperative complications (*p* < 0.0001). Perhaps patients with major complications delayed or were unable to undergo chemotherapy. Major complications may therefore be considered as a strong risk factor for decreased OS and DFS in stage II patients. Additional studies with larger, multicenter, randomized prospective cohorts should be performed to verify these findings.

Given the fact that complications significantly compromise postoperative survival, we further identified factors that increase risk for complications by analyzing patient characteristics and perioperative parameters. However, in our database, the only adjustable factor identified was BMI. Operation time and combined organ resection were mainly associated with tumor stage and technical difficulty. In such circumstances, the prevention and early diagnosis of complications are of critical importance.

The limitations of the present study were its retrospective nature and relatively small sample population. There was a lack of information pertaining to postoperative chemotherapy, which may have affected patient overall survival, but lack of information about chemotherapy maybe didn’t affect the DFS outcomes. The reason that all the patients received standard radical D2 gastrectomy and majority of patients received standard chemotherapy after surgery, the chemotherapy drugs include Oxaliplatin, Xeloda, S-1, Paclitaxel, etc., there maybe no treatment changed before tumor relapse. In our findings, we found the 3-year disease-free survival rate is significantly better in the group of patients without complications than in the group with complications, this trend was also observed in the major or infectious complications group. Further subgroup analysis showed that, among stage II patients, major complications were associated with poorer 3-year DFS rates, Despite these limitations, we believe that major complications are an important and adverse prognostic indicator in patients with gastric cancer.

## Conclusion

Our data confirmed a survival disadvantage in patients with postoperative complications after gastric cancer surgery, with the effect being more profound for cases with major and/or infectious complications. Prevention and early diagnosis of complications are essential to minimize the influence of complications on surgical safety and patient survival.

## Data Availability

The datasets used and analysed during the current study are available from the corresponding author on reasonable request.

## Abbreviations

BMI: Body mass index
DFS: Disease-free survival
OS: Overall survival
